# Transcutaneous Delivery of Immunomodulating Pollen Extract-Galactomannan Conjugate by Solid-in-Oil Nanodispersions for Pollinosis Immunotherapy

**DOI:** 10.3390/pharmaceutics11110563

**Published:** 2019-10-30

**Authors:** Qingliang Kong, Kouki Higasijima, Rie Wakabayashi, Yoshiro Tahara, Momoko Kitaoka, Hiroki Obayashi, Yanting Hou, Noriho Kamiya, Masahiro Goto

**Affiliations:** 1 Department of Applied Chemistry, Graduate School of Engineering, Kyushu University, Fukuoka 819-0395, Japan; kong.qingliang.445@s.kyushu-u.ac.jp (Q.K.); higasijima.kouki.120@s.kyushu-u.ac.jp (K.H.); rie_wakaba@mail.cstm.kyushu-u.ac.jp (R.W.); ytahara@mail.doshisha.ac.jp (Y.T.); mkitaoka@mail.cstm.kyushu-u.ac.jp (M.K.); obayashi.hiroki.495@s.kyushu-u.ac.jp (H.O.); kamiya.noriho.367@m.kyushu-u.ac.jp (N.K.); 2 School of Pharmacy, Shenyang Pharmaceutical University, No.103, Wenhua Road, Shenyang 110016, China; hou.yanting.636@s.kyushu-u.ac.jp; 3 Advanced Transdermal Drug Delivery System Center, Kyushu University, Fukuoka 819-0395, Japan;; 4 Center for Future Chemistry, Kyushu University, Fukuoka 819-0395, Japan

**Keywords:** Japanese cedar pollinosis, Nanocarrier, Pollen extract-galactomannan conjugate, Solid-in-oil nanodispersions, Transcutaneous immunotherapy, Vaccine

## Abstract

Japanese cedar pollinosis is a type I allergic disease and has already become a major public health problem in Japan. Conventional subcutaneous immunotherapy (SCIT) and sublingual immunotherapy (SLIT) cannot meet patients’ needs owing to the side effects caused by both the use of conventional whole antigen molecules in the pollen extract and the administration routes. To address these issues, a surface-modified antigen and transcutaneous administration route are introduced in this research. First, the pollen extract (PE) was conjugated to galactomannan (PE-GM) to mask immunoglobulin E (IgE)-binding epitopes in the PE to avoid side effects. Second, as a safer alternative to SCIT and SLIT, transcutaneous immunotherapy (TCIT) with a solid-in-oil (S/O) nanodispersion system carrying PE-GM was proposed. Hydrophilic PE-GM was efficiently delivered through mouse skin using S/O nanodispersions, reducing the antibody secretion and modifying the type 1 T helper (Th1)/ type 2 T helper (Th2) balance in the mouse model, thereby demonstrating the potential to alleviate Japanese cedar pollinosis.

## 1. Introduction

Japanese cedar (JC) pollinosis is allergic rhinitis, classified to a type I allergic disease that afflicts up to 26.5% of people in Japan [1]. Symptomatic therapy, including pollen avoidance by wearing a mask and glasses, and medical therapy by inhibition of the chemical mediator, are commonly used. In addition, antigen-specific immunotherapy (AIT) is the only available curative treatment for pollinosis, inducing clinical tolerance [2,3]. As a classic form of AIT, subcutaneous immunotherapy (SCIT), which entails repeated injections of increasing doses of the JC pollen extract (PE), has been used in the clinical treatment of pollinosis for over 100 years. Recently, the sublingual application (sublingual immunotherapy, SLIT) of PE has been introduced in the clinical treatment of pollinosis [1]. However, SCIT and SLIT limit the broad applicability of AIT because of the side effects from both conventional antigens of JC PE and administration routes.

JC PE has a long history of use as the antigen for the treatment of pollinosis in Japan. Crude PE has been available since 1969, and standard PE for SCIT and SLIT became available in 2000 and 2014, respectively [4]. Although PE has been proven to induce antigen-specific immune tolerance in a clinical study effectively, there are still some concerns about its safety [5]. All SCIT and the first SLIT must be conducted in the physician’s office. Moreover, all SCIT and SLIT must be under the supervision of medical professionals due to the risk of severe immunoglobulin E (IgE)-mediated adverse events, including anaphylaxis or even death from PE [6]. In the body of an allergic patient, PE can cross-link receptor-bound allergen-specific IgE antibodies on the surface of mast cells and induce an antigen-antibody reaction causing anaphylaxis. To reduce the side effects of PE, several antigen systems for treatment of pollinosis were developed, including T cell epitope peptides and a modified antigen. Antigen-derived T cell epitope peptides are short, without a conformational structure; therefore, they fail to cross-link receptor-bound IgE antibodies [7]. The treatment of pollinosis with T cell epitope peptides could reduce the side effects of PE. Some literature has already reported the therapeutic effect of T cell epitope peptides derived from JC pollen allergen (*Cryptomeria japonica,* Cry j 1 and Cry j 2) for the treatment of pollinosis [7,8,9,10]. Another approach uses a modified antigen, inwhich the IgE-binding epitopes in PE are masked by the attachment of polysaccharides. The attachment of galactomannan (GM) using the Maillard reaction was successfully used to mask the IgE-binding epitopes in PE [11,12]. The binding of the patient’s sera IgE to PE was completely inhibited in conjugation with GM. The recent study has shown that oral administration of antigen-GM conjugate was effective and induced immune tolerance of pollinosis [13]. Therefore, PE-GM conjugate is considered a new antigen for the safe treatment of pollinosis. 

Another concern about conventional AIT of pollinosis is the administration route. Since SCIT and SLIT require lengthy treatments of PE of at least three years [1], the pain associated with SCIT and adverse events from SLIT (local itching and swelling) significantly decrease the levels of patient compliance and persistence [14,15,16]. Transcutaneous immunotherapy (TCIT), as an alternative to SCIT and SLIT, is safe, noninvasive, and cost-effective [17]. Antigen-presenting cells (APCs), which include Langerhans cells and dermal dendritic cells (DCs) in the skin, play central roles in the induction of immunity [18,19]. However, well-functioning skin prevents the intrusion of extraneous molecules and organisms; in particular, the hydrophobic property of the topmost layer of the skin, the stratum corneum (SC), acts as a strong barrier against relatively large hydrophilic antigens (over 500 Da) such as peptides and proteins [20,21]. To overcome this issue, solid-in-oil (S/O) nanodispersions were proposed. S/O nanodispersions are composed of nanosized particles of a hydrophilic antigen coated by a hydrophobic surfactant molecule dispersed into an oil vehicle [22,23]. In previous studies, peptides, as well as proteins, were encapsulated into S/O nanodispersions and penetrated the hydrophobic SC assisted by surfactants and an oil vehicle [24,25,26,27]. Although our previous studies reported the TCIT of pollinosis using T cell epitope peptides [8,27], no study has focused on the transcutaneous delivery of modified antigen PE-GM for TCIT of pollinosis.

**Figure 1:**
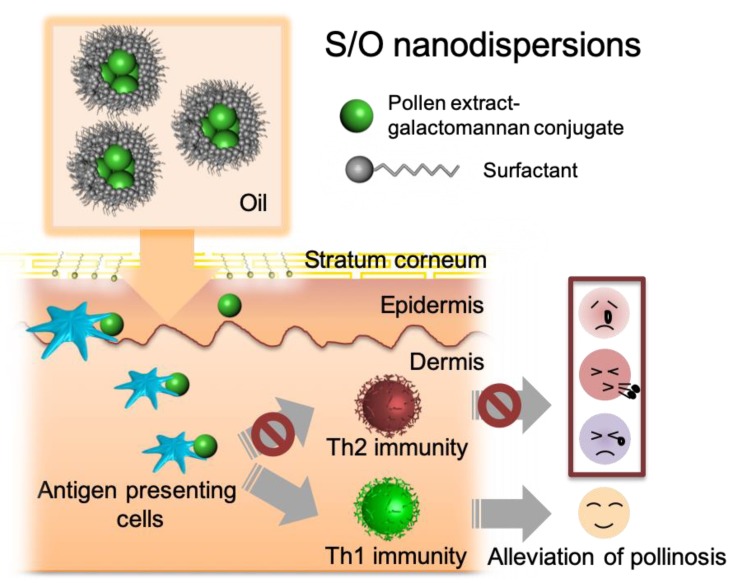
Graphical abstract of this study.

Here, the potential of TCIT using S/O nanodispersions carrying PE-GM was investigated (Figure 1). PE and PE-GM were encapsulated in the S/O nanodispersions, after which the release efficiency and skin permeability of PE and PE-GM were examined using in vitro and in vivo techniques. The difference between PE and PE-GM uptake by DCs was measured. Finally, we evaluated whether TCIT with S/O nanodispersions carrying PE-GM could achieve a similar therapeutic effect of pollinosis compared with that of subcutaneous injection. Our data reveal that TCIT using S/O nanodispersions carrying PE-GM induced the increase and decrease of type 1 T helper (Th1) and type 2 T helper (Th2) immunity, respectively, and PE-GM functioned as an immune response modifier.

## 2. Materials and Methods 

### 2.1. Materials

Cedar pollen extract (PE) and pollen extract-galactomannan conjugate (PE-GM) were purchased from Wako Filter Technology Company (Tokyo, Japan). Fluorescein-4-isothiocyanate (FITC) was purchased from Dojindo (Kumamoto, Japan). Cyclohexane and isopropyl myristate (IPM) were obtained from Wako Pure Chemical Industries (Kyoto, Japan) and Tokyo Chemical Industry (Tokyo, Japan), respectively. A surfactant sucrose laurate (L-195) was kindly provided by Mitsubishi–Kagaku Foods (Tokyo, Japan). RPMI-1640 medium, fetal bovine serum (FBS), antibiotic-antimycotic solution, and Imject Alum were from Thermo Fisher Scientific (Waltham, MA, USA). Histamine dihydrochloride was provided by Nacalai Tesque (Kyoto, Japan). Biotin-conjugated Cry j 1 was obtained from Funakoshi (Tokyo, Japan). Horseradish peroxidase-labeled rabbit anti-mouse IgG 1, and IgG 2a were obtained from Rockland Immunochemicals (Gilbertsville, PA, USA). All other reagents used in the experiments were of analytical grade.

### 2.2. Animals

Female B10.S mice (5–6 weeks old) were purchased from Japan SLC (Shizuoka, Japan) a week prior to experimentation, and housed under standard conditions. Animal experiments were carried out with the approval of the Ethics Committee for Animal Experiments, Kyushu University (approval no. A30-277-0 Date: 2018.10.16), and in accordance with the Guide for the Care and Use of Laboratory Animals (Science Council of Japan).

### 2.3. Preparation of the S/O Nanodispersions

The S/O nanodispersions were prepared according to a previously described method (Figure S1) [27]. Briefly, an aqueous solution of antigen (PE or PE-GM, 0.5 mg/mL, 2 mL) and a cyclohexane solution of sucrose laurate L-195 (12.5 mg/mL, 4 mL) were homogenized with a polytron homogenizer (Kinematica AG, Luzern, Switzerland) at 26,000 rpm for 2 min to form a water-in-oil (W/O) emulsion. The W/O emulsion was flash-frozen in liquid nitrogen for 20 min, and then lyophilized for 24 h with a lyophilizer FDU-1200 (Eyela, Tokyo, Japan). IPM (1 mL) was added to the resultant viscous surfactant-antigen complex to obtain an S/O nanodispersion. The average diameter and polydispersity index of the particles were analyzed with a Zetasizer Nano ZS light scattering instrument (Malvern, Worcestershire, UK). The S/O nanoparticle morphology was analyzed by scanning electron microscopy (SEM) using a Helios NanoLab 600i system (FEI, Hillsboro, OR, USA). S/O nanodispersions were prepared using cyclohexane and then drop-cast on a scanning transmission electron microscope (STEM) grid, washed with cyclohexane and dried under ambient conditions. The specimen was then sputter-coated with platinum (MSP-1S, Vacuum Device, Ibaraki, Japan) and imaged by SEM.

### 2.4. In Vitro Analysis of the Antigen Release from the S/O Nanodispersions

Antigen release tests were performed using custom-fabricated Franz diffusion cells (effective diffusion area: 0.785 cm^2^, receptor volume: 5 mL). Labeled S/O nanodispersions were prepared from PE or PE-GM labeled with FITC (FITC-PE or FITC-PE-GM). A polycarbonate membrane (Avanti Polar Lipids, Inc., Alabaster, AL, USA) was set on a cell, and the receptor compartment was filled with PBS. S/O nanodispersions loaded with FITC-PE or FITC-PE-GM were placed on the membrane and the cell was incubated for 48 h at 37 °C. Samples were collected from the receptor compartment at 1, 2, 4, 8, 24, and 48 h and replaced with the same volume of fresh media. The release of the FITC-labeled antigen was evaluated using a fluorescence spectrometer LS-55 (PerkinElmer; Waltham, MA, USA).

### 2.5. In Vitro Analysis of the Skin Permeation of the Antigen by the S/O Nanodispersions

The mouse back skin was used to examine the skin permeation of FITC-PE or FITC-PE-GM applied via the S/O nanodispersions. The skin was cut into 2 cm^2^ pieces and set onto Franz diffusion cells. S/O nanodispersions and PBS solutions were placed on the surfaces of the skins, and the cells were incubated for 6 h at 32.5 °C. After incubation, the skins were washed with PBS, methanol, and acetonitrile (2:1:1, *v/v/v*) extraction solution to remove the antigen on the skin surface. To determine the cumulative amounts of FITC-PE or FITC-PE-GM in the skin, the skins were cut and subjected to extraction in the extraction solution by vortexing for 12 h. The FITC-PE and FITC-PE-GM concentrations were measured with a fluorescence spectrometer LS-55.

### 2.6. In Vivo Analysis of the Skin Permeation of Antigen by S/O Nanodispersions

Hand-made gauze patches (approximately 0.5 × 1.0 cm^2^) immersed in PBS solution or S/O nanodispersions with FITC-PE or FITC-PE-GM were administered on the ear auricles of mice (1 mg/mL, 50 µg PE, or PE-GM/mouse). After 24 h, the patches were removed, the mice were sacrificed, and the ear auricles were harvested and washed with ethanol and PBS. The earpieces were embedded in optimal cutting temperature (O.C.T.) compound (Sakura Finetek, Tokyo, Japan) and frozen in liquid nitrogen. The frozen skin sections (20 μm) were prepared using a CM1510 cryostat microtome (Leica, Wetzlar, Germany), and imaged on a fluorescence microscope BZ-9000 (Keyence, Osaka, Japan).

### 2.7. Antigen Uptake by DC2.4 Cells

DC2.4 cells were grown in RPMI-1640 medium consisting of 10% heat-inactivated fetal bovine serum (FBS), antibiotic-antimycotic solution, 0.05 mM 2-mercaptoethanol, 10 mM N-(2-hydroxyethyl)piperazine-N’-2-ethanesulfonic acid (HEPES), and 1% non-essential amino acid (NEAA). In the uptake assays, the cells were seeded at a density of 4 × 10^5^ cells per well in the dish (Thermo Fisher Scientific) one day prior to the treatment. FITC-PE or FITC-PE-GM was added to DC2.4 cells at a final concentration of 0.2 mg/mL, and cells were incubated at 37 °C for 24 h. The uptake of FITC-PE or FITC-PE-GM was then immediately determined by flow cytometry (Sony, ec800, Tokyo, Japan) and expressed as mean fluorescence intensity (MFI). At least 15,000 cells per sample were analyzed.

### 2.8. Confocal Imaging of DC2.4 Cells

DC2.4 cells were grown in RPMI-1640 medium, as outlined in Section 2.7. The cells were seeded at a density of 2 × 10^4^ cells per well in a glass-bottom dish (Matsunami, Osaka, Japan) one day prior to the treatment. FITC-PE or FITC-PE-GM was added to the DC2.4 cells at a final concentration of 0.2 mg/mL. The cells were then incubated at 37 °C for 24 h prior to staining with Lysotracker Red (Thermo Fisher Scientific) and fixation with 4% formaldehyde. Finally, cells were imaged by an LSM 700 confocal laser-scanning microscope (Carl Zeiss, Jena, Germany). Negative controls (cells labeled with the Lysotracker Red only) were used to set up the imaging conditions. Colocalization and image analysis were done using the LSM 700 image program.

### 2.9. Sensitization

Mice were sensitized to the PE according to our previous research [27]. Briefly, mice were sensitized with a mixture of PE (10 µg) and Imject Alum (4 mg) by subcutaneous injection once a week for three weeks. Six days after the final subcutaneous injection, histamine dihydrochloride in PBS (2 µg/mL) was dropped into each nostril (5 µL each). Subsequently, PE diluted with PBS (100 mg/mL) was challenged into each nostril (5 mL) for five days from the day after the histamine administration. Blood samples were collected three days after the final challenge. The total antibody IgE levels were measured to determine the pollinosis mouse model. Mice showing high levels of total IgE in sera were considered for the pollinosis model and were subjected to the treatments.

### 2.10. TCIT Treatment of the PE and PE-GM

Hand-made gauze patches immersed in S/O nanodispersions carrying the PE or PE-GM were administered to the ear auricles of the pollinosis mouse model (1 mg/mL, 50 µg PE, or PE-GM/mouse) once a week for three consecutive weeks (Table S1). The patches were covered with surgical adhesive tape and removed 48 h later. Mice treated with PE and Imject Alum by subcutaneous injection instead of treatment were used as a control and followed the same schedule. Six days after the final administration, histamine dihydrochloride in PBS (2 mg/mL) was administrated into each nostril (5 mL each). Subsequently, PE diluted with PBS (100 mg/mL) was dropped into each nostril (5 mL each) for five consecutive days. Blood samples were collected for serum three days after the final PE challenge. One week after the final PE challenge, the mice were sacrificed, and the spleens were harvested for use in cytokine assays. Each group consisted of nine mice.

### 2.11. TCIT Treatment of the Pollinosis Mouse Model Using PE-GM

Hand-made gauze patches immersed in S/O nanodispersions loaded with PE-GM were administered on the ear auricles of pollinosis model mice (1 mg/mL, 50 µg PE-GM/mouse) once a week for three consecutive weeks (Table S2). As a positive control, PE-GM in PBS solution was subcutaneously injected following the same schedule used for the TCIT. Mice treated with PE and Imject Alum by subcutaneous injection instead of treatment using the same schedule were used as a control. The treatment of histamine dihydrochloride and PE, and the collection of serum and spleens were the same as in Section 2.10.

### 2.12. Measurement of the Serum Antibody Levels

Total IgE and Cry j 1-specific IgE were determined with an IgE Mouse Uncoated ELISA Kit (Thermo Fisher Scientific) as previously described [27]. Cry j 1-specific IgG1 and IgG2a levels were also measured by enzyme-linked immunosorbent assay (ELISA). Serum standards were collected from the mice subjected to injection of PE and Imject Alum, while the other mice received TCIT or SCIT. Levels of Cry j 1- specific IgE, Cry j 1- specific IgG1 and IgG2a in standard serum were assigned an arbitrary value of 10,000 relative units (RU)/mL. The data are expressed below as the mean ± standard deviation of *n* = 9.

### 2.13. Cytokine Measurement

Spleen cell mixtures from nine mice per group (1 × 10^7^/well) were incubated in a 96-well plate with 100 µg of PE for 72 h (5% CO_2_, 37 °C). The supernatant cell culture media was then collected, and the cytokine profile of the culture supernatant was measured using ELISA kits from Thermo Fisher Scientific. The data presented were the mean ± standard deviation of quadruplicate tests. 

### 2.14. Effect on Pollen-Induced Nose Rubbing

After the last intranasal pollen extract challenge, the number of nose rubs was measured for 5 min using video recordings.

### 2.15. Statistical Tests

The data were expressed as the mean ± standard deviation (S.D.). Statistical analysis was performed using t-test or one-way analysis of variance (ANOVA), conducted with Graph Pad Prism 6 (GraphPad Software, La Jolla, CA, USA). The level of significance was * *p* < 0.05, ** *p* < 0.01, *** *p* < 0.001, **** *p* < 0.0001.

## 3. Results and Discussion

### 3.1. Preparation and Characterization of S/O Nanodispersions Carrying PE or PE-GM

**Figure 2:**
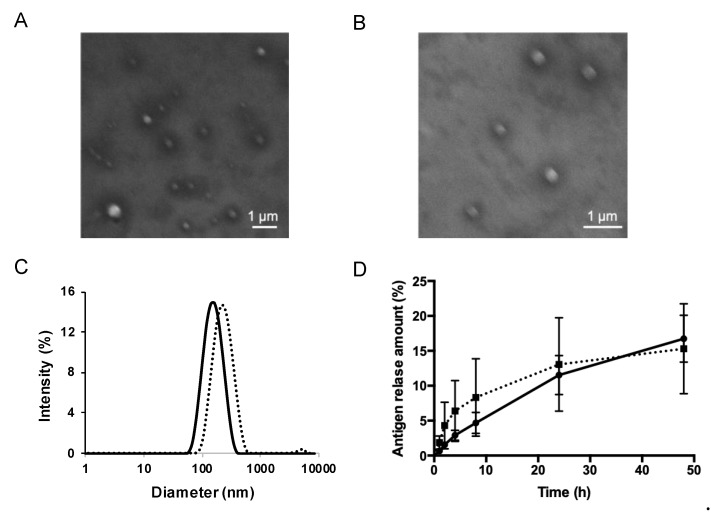
Characterization of solid-in-oil (S/O) nanodispersions. SEM images of S/O nanoparticles loaded with (**A**) pollen extract (PE) and (**B**) pollen extract-galactomannan conjugate (PE-GM). (**C**) Particle size distribution in PE-S/O nanoparticles (solid line) and PE-GM-S/O nanoparticles (dotted line) nanodispersions. (**D**) Release of PE (solid line) and PE-GM (dotted line) from the S/O nanodispersions. *n* = 4, mean ± SE.

PE and PE-GM, as the antigens of pollinosis, were encapsulated into S/O nanodispersions. The materials prepared for S/O nanodispersions, including the surfactant sucrose laurate (L-195) and oil vehicle IPM, are widely used as cosmetics, pharmaceuticals, and food products. S/O nanodispersions carrying PE or PE-GM was transparent and homogeneous. Nanosized particle formation was confirmed by morphological observation using SEM and size distribution analysis using dynamic light scattering. Spherical structures of the surfactant-antigen complex were observed by SEM (Figure 2A,B). The average particle sizes were 163.9 and 212.8 nm for the S/O nanodispersions carrying PE and PE-GM, respectively (Figure 2C, Table 1). These results indicated that the nanosized antigen-surfactant complex particles were successfully dispersed in the IPM, if PE or PE-GM were used, which contains not only proteins but also large molecular weight materials, as core hydrophilic drugs in the S/O formulation.

The antigen release efficiency from S/O nanodispersions was evaluated using a Franz diffusion cell instrument. The release efficiency of antigens from S/O nanodispersions is one of the most important factors that influence vaccination efficiency. Both PE and PE-GM in S/O nanodispersions could be released from the S/O formulations (Figure 2D). We confirmed that the antigen release efficiency from the tested S/O nanodispersions was not affected by the modification of PE using GM. The result concurred with a previous report demonstrating that S/O nanodispersions became unstable after contacting with water, causing the release of the encapsulated antigen [25]. The encapsulation efficiency of the antigen in the carrier is also crucial for the vaccine. We previously reported a high antigen encapsulation efficiency of a peptide in S/O nanodispersions up to 99.5% [25]. S/O nanodispersions do not require a specific interaction between encapsulating the antigen and surfactant and, therefore, could also have a high encapsulation efficiency of hydrophilic antigens, including PE and PE-GM.

### 3.2. Skin Permeation by S/O Nanodispersions Carrying PE or PE-GM

**Figure 3:**
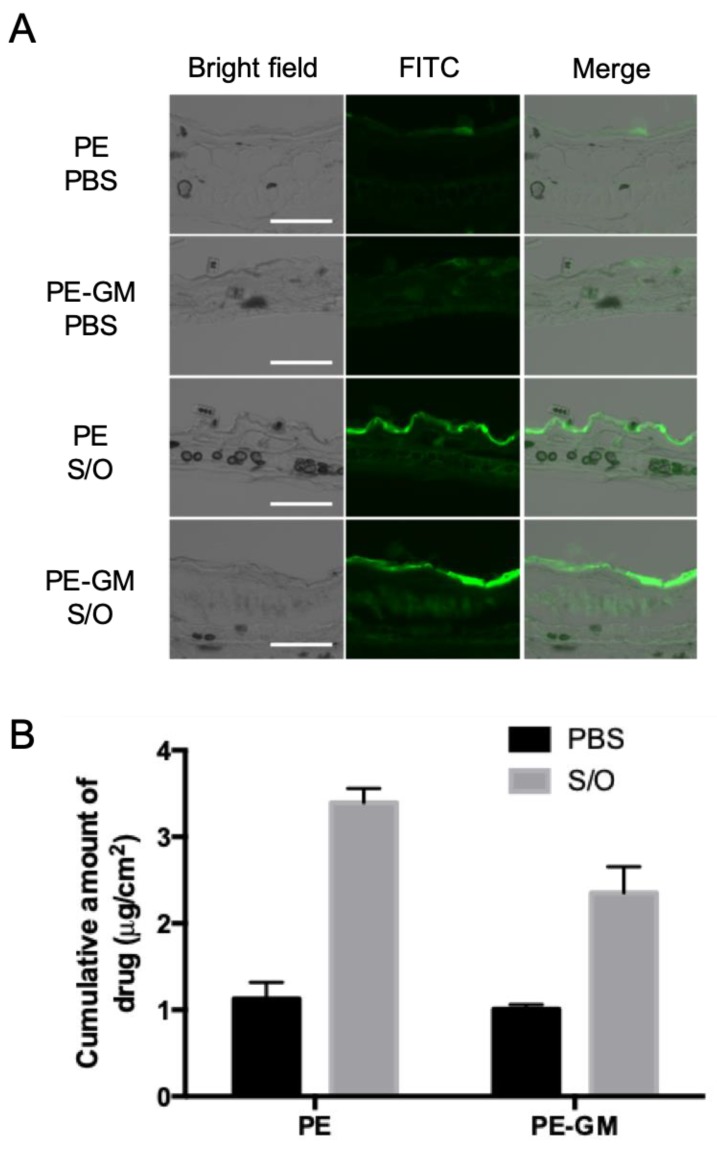
Transcutaneous delivery of antigen to the skin by S/O nanodispersions. (**A**) Skin sectioning fluorescence microscopy images of PE and PE-GM permeating the mouse-ear auricle skin in vivo and (**B**) the cumulative amount of PE and PE-GM in the skin following 6 h incubation in vitro with a PE or PE-GM PBS solution (PBS) and S/O nanodispersions (S/O). Bars: 100 μm. *n* = 3–4, mean ± SE.

The skin permeation was evaluated using FITC-labeled antigens FITC-PE and FITC-PE-GM. S/O nanodispersions or PBS solution of FITC-PE and FITC-PE-GM were applied to the intact ear auricle and back skin from mice using hand-made patches. As shown in Figure 3A, mouse-ear auricle sections processed with a FITC-PE or FITC-PE-GM PBS solution showed a weak fluorescence intensity on the top layer of the skin. In contrast, sections treated with the S/O nanodispersions exhibited a strong fluorescence intensity. Similar images of sections from the back skin were observed (Figure S2), suggesting the enhanced permeation of antigens into the skin after applying S/O nanodispersions.

The cumulative amount of antigen permeating the skin was examined using the mouse skins onto Franz diffusion cells and determined by extracting the antigen from the skin. Notably, the amount of FITC-PE that permeated the skin was three times greater for the S/O nanodispersions than that for the PBS solution (Figure 3B). The S/O nanodispersions facilitated the permeation of the FITC-PE-GM by 2.3-fold compared with that of the PBS solution. The cumulative amount of antigen that permeated the skin was concordant with the imaging results.

In this study, most of the antigen remained in the SC layer (Figure 3A, Figure S2). Our previous study suggested that the SC layer could act as a reservoir for S/O nanodispersions to help the antigen sustain release [8,25]. The main mechanisms of antigen permeation and the permeation route were previously discussed, suggesting that IPM is a skin penetration enhancer, allowed the intercellular permeation of S/O nanoparticles by increasing fluidity of intercellular lipids in the SC.

### 3.3. Antigen Uptake by DC2.4 Cells

**Figure 4:**
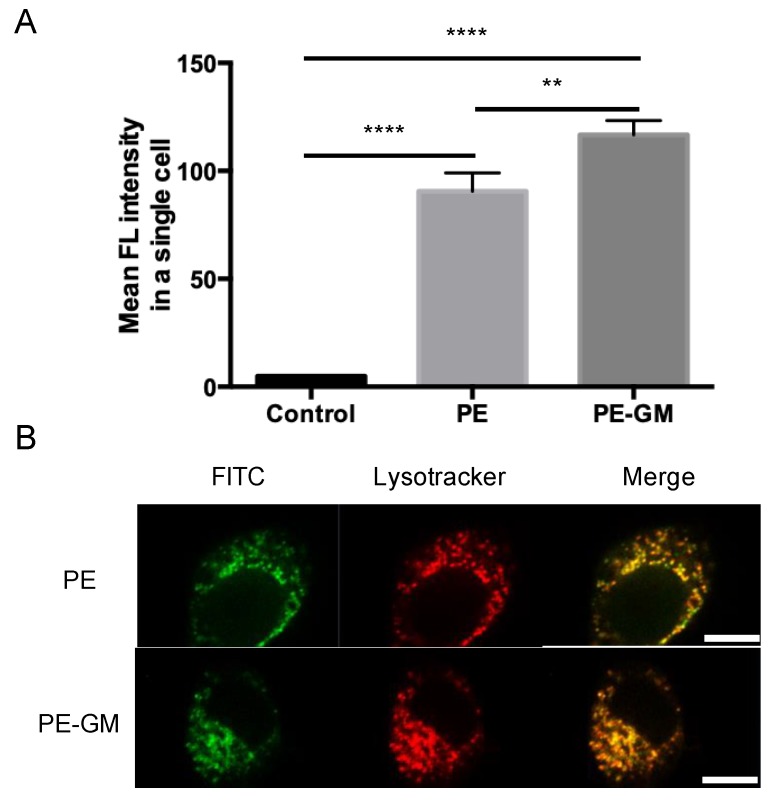
(**A**) Comparison of the mean fluorescent intensity of non-treated cells (control), cells treated with FITC-labeled PE, and PE-GM. (**B**) Confocal images of dendritic cells (DCs) treated with FITC labeled PE and PE-GM. Lysosomes at DCs were stained with Lysotracker (red). Bars: 10 μm. *n* = 3, mean ± SE. ** *p* < 0.01, **** *p* < 0.0001.

To understand the difference of PE before and after modification by GM, PBS solution of FITC-PE and FITC-PE-GM was applied to DC2.4 cells. The uptake of FITC-PE or FITC-PE-GM by DC2.4 cells was determined by flow cytometry and expressed as mean fluorescence intensity. The amount of antigen uptake by DC2.4 cells was 1.3 times greater for FITC-PE-GM than FITC-PE (Figure 4A). The result revealed that FITC-PE-GM could be taken by DCs more efficiently than FITC-PE. Moreover, we focused on the pathway of antigen uptake and performed a colocalization study of the antigen with the Lysotracker dye. The result revealed that both FITC-PE and FITC-PE-GM penetrated the cells and fused with the lysosome, as confirmed by yellow-colored merged images (Figure 4B). A greater colocalization rate of FITC-PE-GM and lysosomes was observed compared with that of FITC-PE (Figure S3). 

The improved uptake of PE-GM may be related to the mannose receptor (MR) in DCs. It was reported that the hypermannosylated antigen was taken up more efficiently by DCs than the natural antigen through the binding to C-type lectin receptors like MR [28]. Both Cry j 1-galactomannan and Cry j 1-mannose were easily caught in DCs compared with Cry j 1 alone [12]. In our system, the PE uptake efficiency by DCs was improved by the modification of PE with GM, leading to subsequent antigen presentation to the T cells.

### 3.4. Comparison of TCIT Using S/O Nanodispersions Carrying PE or PE-GM

**Figure 5:**
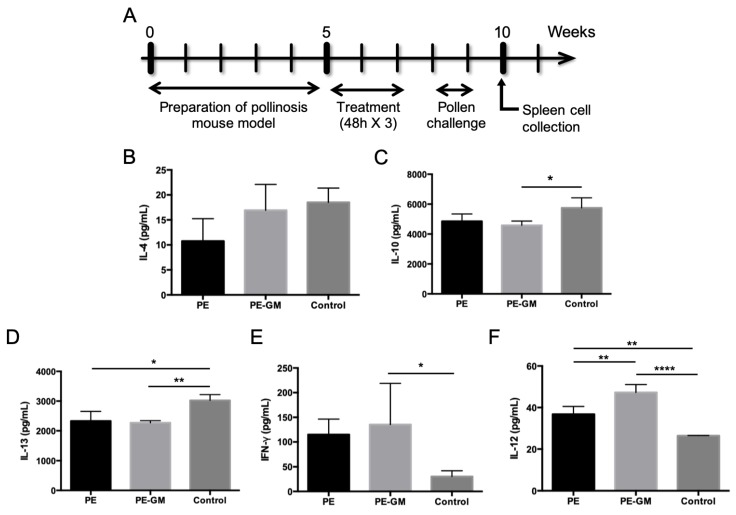
(**A**) The schedule of sensitization and immunotherapy. Levels of (**B**) IL-4, (**C**) IL-10, (**D**) IL-13, (**E**) IFN-γ and (**F**) IL-12 allergen-induced type 1 T helper (Th1) and type 2 T helper (Th2) cytokines produced by splenic cells isolated from mice as measured by enzyme-linked immunosorbent assay (ELISA). Splenocytes were isolated from B10.S mice immunized by the transcutaneous administration of PE and PE-GM in S/O nanodispersions. Splenocytes were stimulated with PE (50 μg/mL) for three days. *n* = 4, mean ± SE. * *p* < 0.05, ** *p* < 0.01, **** *p* < 0.0001.

Sensitization and immunotherapy were performed following the above-described schedule (Figure 5A). Mice were treated with the patch containing S/O nanodispersions carrying PE or PE-GM (Table S1). The total IgE level is often used as a marker of pollinosis because IgE antibody secretion is closely related to allergic reactions. After TCIT via the S/O nanodispersions carrying PE or PE-GM, the total IgE levels decreased compared with the control group (Figure S4), indicating that both PE and PE-GM were able to suppress the allergic immune response. Cry j 1-specific IgG1 and IgG2a were measured to evaluate the balance of Th1/Th2 immunity. A reduction in the ratio of Cry j 1-specific IgG1 to IgG2a antibodies was observed in the mice sera treated with the S/O nanodispersions carrying PE-GM, indicating that Th2-dominant immunity was skewed to Th1-dominant immunity by PE-GM, although none of these differences achieved statistical significance (Figure S5). The profile of cytokines secreted by splenocytes from the mice described above was examined (Figure 5B-F). After TCIT via the S/O nanodispersions, Th2 type cytokine levels (IL-4, IL-10, and IL-13) were decreased; however, Th1 type cytokine levels (IFN-γ and IL-12) increased. Especially in the splenocytes from mice treated with TCIT of S/O nanodispersions containing PE-GM, levels of IL-10, IL-12, IL-13, and IFN-γ cytokines showed significant changes compared with those from the mice treated with the control solution. Moreover, a significant increase in the IL-12 level was observed by the treatment with S/O nanodispersions carrying PE-GM compared with that carrying PE.

Although some literature reports that the attachment of galactomannan to an antigen is efficiently caught by DCs, to the best of our knowledge, few research studies discussed the details of antigen-galactomannan fate in immune responses after antigen uptake of DCs. After PE-GM was taken by the DCs and presented to the T cell, the T cell differentiation toward Th1 and Th2 shifted to better balance. Enhanced Th1 and reduced Th2 populations were suggested through the change of cytokines secreted by antigen-stimulated cultured splenocytes treated with PE-GM. Similar changes were reported in the other antigen glycoconjugates, such as Fag e 1-glucomannan conjugate and ovalbumin-mannose conjugate. Oral administration of Fag e 1-glucomannan conjugate caused marked differentiation for the increase of Th1 and decrease of Th2 [29]. Oral administration of ovalbumin-mannose conjugate was reported to alleviate an egg allergy and the changes in Th1 type cytokine IL-12 and Th2 type cytokine IL-4 and IL-17 better regulated cytokine production toward a balanced response than those otherwise treated [30]. These results supported our finding that PE-GM showed an improvement in the Th1/Th2 balance in the pollinosis mouse model, suggesting that PE-GM could be available for an immunomodulating antigen for the treatment of pollinosis.

### 3.5. Comparison of the Differences between TCIT and SCIT Using PE-GM

**Figure 6:**
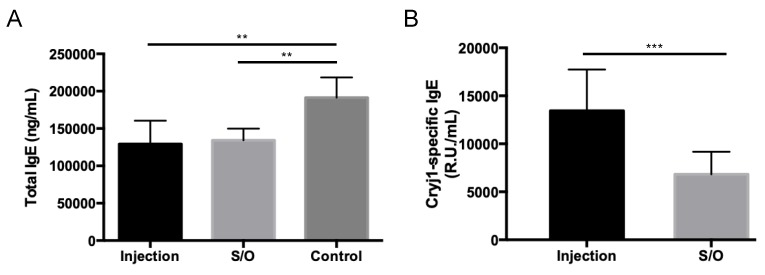
Serum antibody responses after immunotherapy using PE-GM in a pollinosis mouse model. PE-GM in S/O nanodispersions (S/O) and PE-GM PBS solution (Injection) were administrated once a week for three weeks, and (**A**) total immunoglobulin E (IgE) and (**B**) Cry j 1-specific IgE levels in the mice were measured by ELISA. *n* = 9, mean ± SE. ** *p* < 0.01, *** *p* < 0.001.

**Figure 7:**
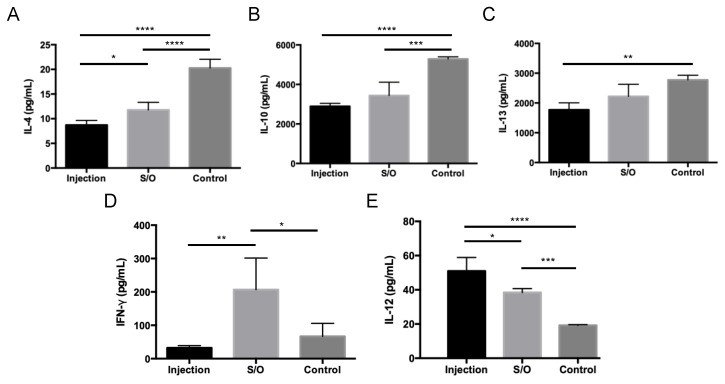
Allergen-induced Th1 and Th2 cytokine production by splenic cells isolated from mice. Levels of (**A**) IL-4, (**B**) IL-10, (**C**) IL-13, (**D**) IFN-γ, and (**E**) IL-12 were measured by ELISA. Splenocytes were isolated from B10.S mice immunized by the transcutaneous administration PE-GM in S/O nanodispersions (S/O) and injection administration PE-GM in PBS solution (Injection). Splenocytes were stimulated with PE (50 μg/mL) for three days. *n* = 4, mean ± SE. * *p* < 0.05, ** *p* < 0.01, *** *p* < 0.001, **** *p* < 0.0001.

Mice were treated with a patch containing S/O nanodispersions carrying PE-GM or subcutaneously injected with PBS solution of PE-GM (Table S2). After treatment via the TCIT or SCIT, the total IgE levels trended lower compared with those in the control group (Figure 6A), indicating that PE-GM was able to suppress the allergic immune response. The Cry j 1-specific IgE level was significantly reduced by the transcutaneous administration of S/O nanodispersions compared with the subcutaneous injection of antigen in a PBS solution (Figure 6B), suggesting that S/O nanodispersions suppressed an allergic immune response more efficiently. The previous study showed that epitope peptides of Cry j 1 were effective for pollinosis immunotherapy with S/O nanodispersions [8,27]. In addition, Figure 6B indicated that PE-GM, which is a crude mixture containing Cry j 1 protein and other macromolecules, was also delivered transcutaneously carrying Cry j 1 antigen by S/O nanodispersions and created antigen-specific immune responses. The profile of cytokines secreted by antigen-stimulated cultured splenocytes from the mice was measured (Figure 7). The decrease of the Th2 type cytokine levels (IL-4, IL-10, and IL-13) and the increase of the Th1 type cytokine levels (IFN-γ and IL-12) were observed by the TCIT using S/O nanodispersions carrying PE-GM. The mice treated with SCIT showed similar results to those treated with TCIT, excepting a low IFN-γ level. As an important clinical symptom of pollinosis, the number of nose rubs was evaluated to investigate the therapeutic effect of TCIT and SCIT. The number of nose rubs in mice treated with TCIT and SCIT was reduced compared to controls (Figure S6). The decrease in antibody levels, cytokine levels, and nose rubs may be related to the successful delivery of PE-GM, which breaks the barrier of SC with the help of the S/O nanodispersions, to the APCs in the epidermis. These results suggested that S/O nanodispersions carrying PE-GM could treat pollinosis effectively and have a similar therapeutic effect compared with the subcutaneous injection of PE-GM PBS solution.

Considering the compliance and persistence of patients, the selection of a better administration route is important for pollinosis immunotherapy because of the long treatment duration. Although SCIT and SLIT are considered the efficient treatments for pollinosis, low compliance and persistence for patients were reported in clinical trials and real-life studies due to the side effects [31]. To address these issues, TCIT was developed to improve compliance and persistence for patients. The T cell epitope peptide mixture and integrated peptide-loaded by S/O nanodispersions that alleviated JC pollinosis were previously reported [27,32]. However, the mechanism of TCIT with different antigen seems different in that T cell epitope peptide caused a decrease of both Th1 and Th2 cytokines, which may be related to the immunosuppressive effect of T regular cells [27]. Current data suggest that S/O nanodispersions loaded with PE-GM induced an increase and decrease in Th1 and Th2 type cytokines, respectively, and PE-GM functioned as an immune response modifier rather than an immunosuppressor.

## 4. Conclusions

We evaluated the effect of transcutaneous delivery of intact pollen extract (PE) and pollen extract-galactomannan conjugate (PE-GM) in S/O nanodispersions on pollinosis immunotherapy. S/O nanodispersions enhanced the skin permeability of both PE and PE-GM. The modification of PE by GM improved the uptake of PE by DCs and showed an improvement in Th1 immunity in a pollinosis mouse model. A modulation of the immune response was observed by the transcutaneous administration of the S/O nanodispersions containing PE-GM, achieving a similar therapeutic effect compared with the injection method. This simple and effective transcutaneous immunotherapy has a high potential as an alternative to subcutaneous administration and sublingual administration for the future treatment of Japanese cedar pollinosis.

## Figures and Tables

**Table 1:** Size analysis of S/O nanoparticles loaded with pollen extract (PE) or pollen extract-galactomannan conjugate (PE-GM).

Sample	Diameter (nm)	PDI
PE-S/O	163.9 ± 0.4	0.181–0.196
PE-GM-S/O	212.8 ± 8.6	0.111–0.238

^1^ PDI = polydispersity index. mean ± SE of *n* = 3.

## Supplementary Materials

The following are available online at https://www.mdpi.com/1999-4923/11/11/563/s1, Figure S1: Preparation of S/O nanodispersions carrying antigen PE or PE-GM. Figure S2: Skin sectioning fluorescence microscopy images of PE and PE-GM permeated into mice back skin in vivo following 24 h incubation with PE and PE-GM PBS solution (PBS), S/O nanodispersions (S/O). Figure S3: Comparison of colocalization of lysosome and antigens after DCs treated with FITC labeled PE and PE-GM. Figure S4: Serum antibody responses after immunotherapy using antigen PE or PE-GM in a pollinosis model mice. Figure S5: Serum antibody responses after immunotherapy using antigen PE or PE-GM in a pollinosis model mice. Figure S6: (A) The number of nose rubs was counted in the 5 min after the last intranasal pollen challenge. (B) Reduced number of nose rubs in pollinosis model mice treated with PE-GM in S/O nanodispersions (S/O) and injection group (Injection). Table S1: Composition of the samples at Materials and Methods section 2.10. Table S2: Composition of the samples at Materials and Methods section 2.11.
