# Supplementary material for: Transcutaneous Delivery of Immunomodulating Pollen Extract-Galactomannan Conjugate by Solid-in-Oil Nanodispersions for Pollinosis Immunotherapy
Source: Pharmaceutics. 2019 Oct 30;11(11):563. doi: 10.3390/pharmaceutics11110563 (PMC6920820; doi:10.3390/pharmaceutics11110563)
Supplement: Supplementary file 1 [file pharmaceutics-11-00563-s001.pdf]

pharmaceutics

Supplementary Materials: Transcutaneous Delivery of Immunomodulating Pollen Extract-Galactomannan Conjugate by Solid-in-Oil Nanodispersions for Pollinosis Immunotherapy

Qingliang Kong, Kouki Higasijima, Rie Wakabayashi, Yoshiro Tahara, Momoko Kitaoka, Hiroki Obayashi, Yanting Hou, Noriho Kamiya and Masahiro Goto

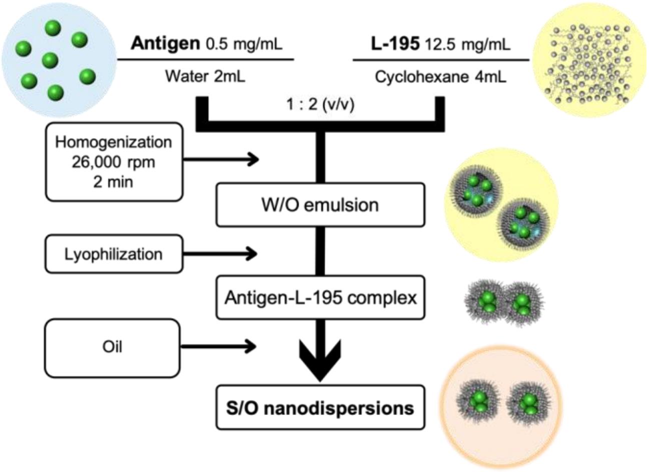

Figure S1. Preparation of S/O nanodispersions carrying antigen PE or PE-GM. A water-in-oil (W/O) emulsion was prepared from an aqueous solution of antigen (PE or PE-GM, 0.5 mg/mL, 2 mL) and a cyclohexane solution of surfactant L-195 (12.5 mg/mL) using a polytron homogenizer at 26,000 rpm for 2 min. The W/O emulsion was flash-frozen in liquid nitrogen for 20 min, and then lyophilized for 24 h with a lyophilizer. Finally, the resultant viscous surfactant-antigen complex was dispersed in isopropyl myristate to yield the S/O nanodispersions.

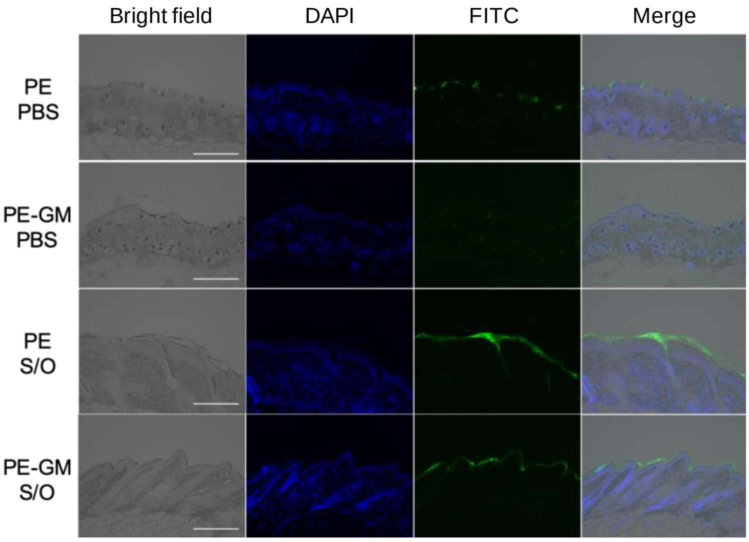

Figure S2. Skin sectioning fluorescence microscopy images of PE and PE-GM permeated into mice back skin in vivo following 24 h incubation with PE and PE-GM PBS solution (PBS), S/O nanodispersions (S/O). Bars: 200 μm.

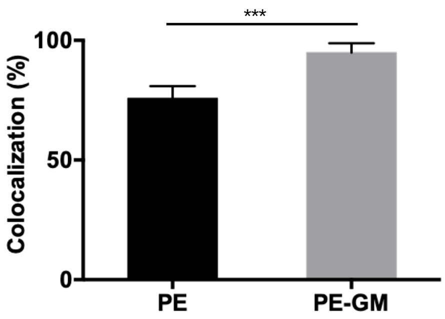

Figure S3. Comparison of colocalization of lysosome and antigens after DCs treated with FITC labeled PE and PE-GM. n = 5, mean ± SE. *** $p < 0.001$.

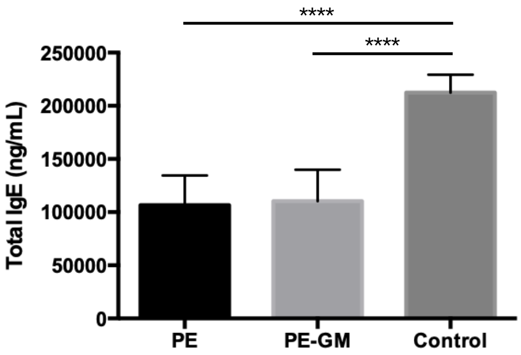

Figure S4. Serum antibody responses after immunotherapy using antigen PE or PE-GM in a pollinosis model mice. Antigen PE or PE-GM in S/O nanodispersions were administrated once a week for 3 weeks, and the serum total IgE level in mice were measured by ELISA. n = 9, mean ± SE. **** $p < 0.0001$.

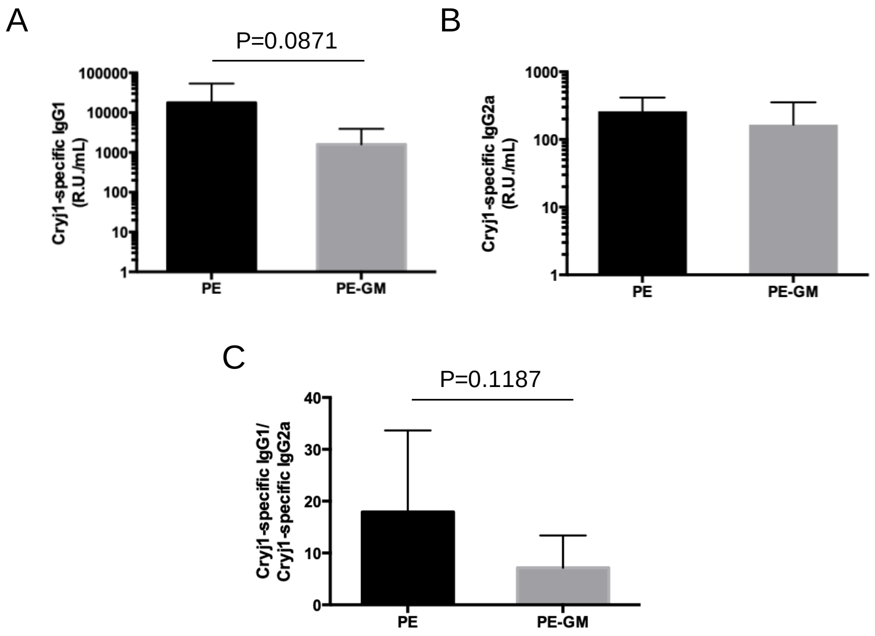

Figure S5. Serum antibody responses after immunotherapy using antigen PE or PE-GM in a pollinosis model mice. Antigen PE or PE-GM in S/O nanodispersions were administrated once a week for 3 weeks, and (**A**) Cry j 1- specific IgG1, (**B**) Cry j 1- specific IgG2a, (**C**) the rate of Cry j 1- specific IgG1 and IgG2a levels in mice were measured. n = 9, mean ± SE.

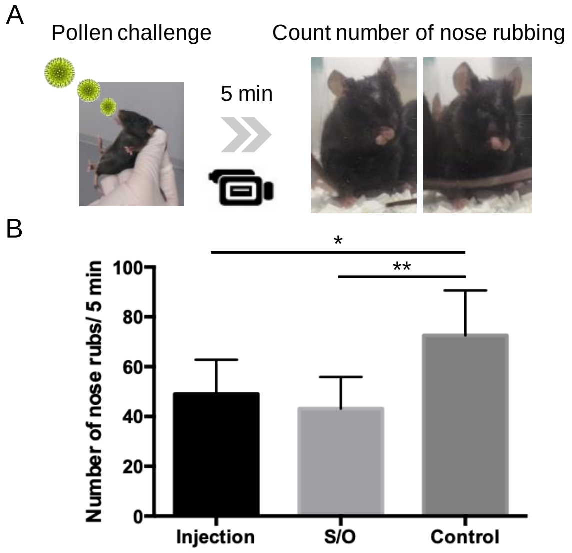

Figure S6. (**A**) The number of nose rubs was counted in the 5 min after the last intranasal pollen challenge. (**B**) Reduced number of nose rubs in pollinosis model mice treated with PE-GM in solid-in-oil nanodispersions (S/O) and injection group (Injection). $n = 9$, mean ± SE. * $p < 0.05$, ** p <0.01.

Table S1. Composition of the samples at Materials and Methods Section 2.10.

**Sample**		Antigen (μg)	L-195 (mg)	IPM (μL)
**Patch**	PE S/O nanodispersions	50	2.5	50
	PE-GM S/O nanodispersions	50	2.5	50

Table S2. Composition of the samples at Materials and Methods Section 2.11.

**Sample**		Antigen (μg)	L-195 (mg)	IPM (μL)	PBS (μL)
**Injection**	PE S/Onanodispersions	50	-	-	50
Patch	PE-GM S/O nanodispersions	50	2.5	50	-

© 2019 by the authors. Submitted for possible open access publication under the terms and conditions of the Creative Commons Attribution (CC BY) license (http://creativecommons.org/licenses/by/4.0/).
